# Endogenous Neurotrophins and Trk Signaling in Diffuse Large B Cell Lymphoma Cell Lines Are Involved in Sensitivity to Rituximab-Induced Apoptosis

**DOI:** 10.1371/journal.pone.0027213

**Published:** 2011-11-04

**Authors:** Cynthia Bellanger, Lydie Dubanet, Marie-Claude Lise, Anne-Laure Fauchais, Dominique Bordessoule, Marie-Odile Jauberteau, Danielle Troutaud

**Affiliations:** 1 Université de Limoges, EA3842, Limoges, France; 2 UMR CNRS 6101, Université de Limoges, Limoges, France; 3 Structure Régionale de Référence des Lymphomes du Limousin, CHU Limoges, Limoges, France; 4 Service d'Hématologie Clinique, CHU Limoges, Limoges, France; Centre de Recherche Public de la Santé (CRP-Santé), Luxembourg

## Abstract

**Background:**

Diffuse large B-cell lymphoma (DLBCL) is a common and often fatal malignancy. Immunochemotherapy, a combination of rituximab to standard chemotherapy, has resulted in improved survival. However a substantial proportion of patients still fail to reach sustained remission. We have previously demonstrated that autocrine brain-derived neurotrophic factor (BDNF) production plays a function in human B cell survival, at least partly via sortilin expression. As neurotrophin receptor (Trks) signaling involved activation of survival pathways that are inhibited by rituximab, we speculated that neurotrophins may provide additional support for tumour cell survival and therapeutic resistance in DLBCL.

**Methodology/Principal Findings:**

In the present study, we used two DLBCL cell lines, SUDHL4 and SUDHL6, known to be respectively less and more sensitive to rituximab. We found by RT-PCR, western blotting, cytometry and confocal microscopy that both cell lines expressed, in normal culture conditions, BDNF and to a lesser extent NGF, as well as truncated TrkB and p75^NTR^/sortilin death neurotrophin receptors. Furthermore, BDNF secretion was detected in cell supernatants. NGF and BDNF production and Trk receptor expression, including TrkA, are regulated by apoptotic conditions (serum deprivation or rituximab exposure). Indeed, we show for the first time that rituximab exposure of DLBCL cell lines induces NGF secretion and that differences in rituximab sensitivity are associated with differential expression patterns of neurotrophins and their receptors (TrkA). Finally, these cells are sensitive to the Trk-inhibitor, K252a, as shown by the induction of apoptosis. Furthermore, K252a exhibits additive cytotoxic effects with rituximab.

**Conclusions/Significance:**

Collectively, these data strongly suggest that a neurotrophin axis, such NGF/TrkA pathway, may contribute to malignant cell survival and rituximab resistance in DLBCL.

## Introduction

Diffuse large B-cell lymphoma (DLBCL) is the most common type of non-Hodgkin's lymphoma (NHL). This lymphoma is a clinically and biologically heterogeneous disease [1]. Rituximab (a chimeric anti-CD20 monoclonal antibody), alone or combined with chemotherapy, has demonstrated efficacy in DLBCL, resulting in an increased proportion of cured patients disease [2]. The *in vivo* mechanisms of rituximab-mediated antitumor effects include antibody-dependent cellular cytotoxicity (ADCC), complement-dependent cell cytotoxicity (CDC), growth-inhibition and apoptosis by binding to the CD20 antigen specifically expressed on B-cells [3]. Constitutively activated growth signaling pathways have frequently been observed in DLBCL tumors and DLBCl cell lines including protein kinase AKT and nuclear factor κB (NF-κB) transcription factor. Activation of these pathways controls a variety of mechanisms that inhibit apoptosis and prolong cell survival notably in resulting downstream overexpression of functional anti-apoptotic proteins such as Bcl-x_L_ and Bcl-2 that can also lead to chemoresistance [4], [5]. Interestingly, by contrast, treatment with rituximab inhibits these survival pathways resulting in augmentation of the association of Bcl-X_L_ and Bad, as well as downregulation of Bcl-X_L_ expression leading to chemosensitization [6]. Despite therapeutic advances, a subset of patients does not respond or relapses after the initial treatment, and the mechanism of rituximab resistance, a growing concern, is not clear [3]. Thus, it is still important to identify new targets for development of additional therapeutic options.

Members of the neurotrophin (NT) family like nerve growth factor (NGF) and brain-derived neurotrophic factor (BDNF) are structurally and functionally related neurotrophic factors that control neuron survival and axon growth during nervous system development and repair [7]. Each NT binds to a specific high-affinity tyrosine kinase receptor, Trk. NGF binds to TrkA, BDNF and NT-4/5 to TrkB. In addition NTs share a common low-affinity p75 NT receptor (p75^NTR^) a member of the tumor necrosis factor (TNF) receptor superfamily. Signaling by NTs through Trk and p75^NTR^ receptors has been extensively studied in the context of nervous system development, survival, and plasticity. NT binding to Trk receptors results in receptor dimerization, leading to subsequent signaling cascades including MAPK (mitogen-activated protein kinase), PI3K/Akt (phosphatidylinositol-3 kinase/Akt) and NF-κB that favour cellular survival [8], [9]. In contrast, the functions of the p75^NTR^ receptor are complex and have been more difficult to ascertain. It is clear that p75^NTR^ functions as a Trk co-receptor that increases binding affinity and specificity of Trk [10], but engagement of p75^NTR^ also triggers divergent pathways involving notably Jun kinase (JNK) activity and caspase activation, that facilitate apoptosis [11]. Thus, while p75^NTR^ binding can increase the survival effect of Trk-NT interactions, p75^NTR^ can also induce apoptosis when Trk is reduced or absent. Furthermore, NTs are synthesized as pro-NTs which proteolysis generates mature NTs. Both the pro-NTs and the mature forms are secreted. However they display opposite biological activities on cell proliferation and apoptosis. Indeed, pro-NTs such as pro-NGF and pro-BDNF, display greater affinity for p75^NTR^ and preferentially interact with sortilin —also known as neurotensin receptor-3 and a member of the Vps10p-domain receptor family —together with p75^NTR^ to form a complex capable of activating an apoptotic cascade [12], [13]. Thus, the overall cellular outcome in response to NT exposure reflects a balance between p75^NTR^ and Trk engagement that is controlled by pro-NT processing.

Although NTs are thought to exert their actions predominantly on neurons, increasing evidence shows that some NTs, notably NGF and BDNF, can also influence the development and activation of many cell types of the immune system, including B lymphocytes [14], [15], [16]. NT (NGF, BDNF) and TrkA, TrkB, p75^NTR^ receptor expressions have been detected in normal human B lymphocytes and in human B cell lines. Membranous or intracellular localization varied according to cell line and culture conditions [17], [18]. In B cells, autocrine BDNF and NGF signaling appears to be essential for the survival of mature and memory B cells, respectively, especially in pro-apoptotic or pro-inflammatory conditions [19], [20], [21]. Trk and their NT ligands have been also found in malignant B cell lines notably multiple myeloma and NHL cell lines [22], [23] but involvement of NTs in the pathogeny of B cell tumors, and notably in DLBCL, needs to be elucidated.

In recent studies we have shown an autocrine production of BDNF and pro-BDNF in human pre-B, mature, and plasmacytic malignant B cell lines. The endogenous BDNF released under stress culture conditions, such as serum deprivation and/or Fas-induced apoptosis, exerts antiapoptotic effects. Furthermore, we demonstrated that this autocrine regulation is linked to the presence of sortilin which has not been previously described in B cells [24]. The goal of this study was to define the NGF and BDNF expression and secretion and their respective TrkA, TrkB and p75^NTR^ receptors in DLBCL cells cultured under standard and apoptotic conditions, including exposure to rituximab, to evaluate a potential link between rituximab sensitivity and NT production. To assess this hypothesis we used two DLBCL cell lines, SUDHL4 and SUDHL6, known to be respectively less and more sensitive to the rituximab induced-apoptosis [25]. We report herein i) that NGF, BDNF, TrkB, p75^NTR^ and its co-receptor sortilin were expressed in two DLBCL cell lines and ii) that their responsiveness to rituximab was depending on NT secretion and TrkA expression suggesting a relationship between Trk signaling and rituximab sensitivity.

## Materials and Methods

### Human B cell lines and cell cultures

The CD20-expressing human DLBCL cell lines SUDHL4 and SUDHL6 were obtained from DSMZ (Braunschweig, Germany). Under basal culture conditions, the B cell lines (5×10^5^ cells/ml) were cultured in RPMI 1640 medium (Gibco, Grand Island, NY, USA) supplemented with 10% heat-inactivated Fetal Calf Serum (FCS), 100 U/ml penicillin, 100 µg/ml streptomycin and 2 mM L-glutamine (Gibco) at 37°C in a humidified atmosphere containing 5% CO_2_. NTs production and expression of p75^NTR^ and Trk receptors were analyzed in different cell culture conditions (24–72 h): with (control) and without FCS (serum deprivation), in the presence of 1–20 µg/ml of rituximab (MabThera®, stock 10 mg/ml, a generous gift from CHRU Dupuytren of Limoges, Pharmacie centrale, France) or control human IgG (Jackson ImmunoResearch Europe Ltd., Newmarket, UK). For K-252a sensitivity tests, cells were grown in triplicate in 96 well tissue culture plates (starting with 5×10^4^ cells/well) with various concentrations of K-252a (0–500 nM, Alomone labs, Jerusalem, Israel).

### Antibodies

Rabbit mAb anti-Akt (Akt[pan], C67E7), anti-phospho-Akt (Ser473, D9E and Thr308, C31E5E), and rabbit mAb anti-β-actin (13E5) were obtained from Cell Signaling Technology (Beverly, MA, USA). Mouse mAbs Anti-TrkA (MAB1751) and anti-TrkB (MAB3971) were purchased from R&D System (Minneapolis, MN, USA). Anti-NGF (rabbit polyclonal Ab, H-20), anti-BDNF (rabbit polyclonal Ab, N-20), anti-p75^NTR^ (H-137, a rabbit polyclonal Ab reactive with the N-terminal domain of p75^NTR^), anti-p75^NTR^ (H-92, a rabbit polyclonal Ab reactive with the C-terminal domain of p75^NTR^) and anti-sortilin (a goat polyclonal Ab, C20) antibodies were purchased from Santa Cruz Biotechnology (Santa Cruz, CA, USA). Purified mouse or rabbit IgG Ab (Sigma Aldrich, Saint Louis, MO, USA) were used as negative controls to determine background and positive thresholds.

### Cell viability assay

Cellular viability of DLBCL cell lines was assessed using the colorimetric XTT (sodium 3′-[1-(phenylaminocarbonyl)-3,4-tetrazolium]-bis(4-methoxy-6-nitro) benzene sulfonic acid hydrate)-assay (Cell Proliferation Xit II, XTT Roche, Meylan, France) according to the manufacturer's instructions. Results are expressed as the relative cell viability of untreated control cells.

### Analysis of apoptosis

DNA fragmentation was detected by flow cytometric analysis after propidium iodide staining as previously described [26] and using a FACSVantage DIVA SE flow cytometer. Percentage of apoptotic cells was then defined as the percentage of cells with DNA staining before G0/G1 peak (hypodiploid, sub-G1 peak). Furthermore apoptosis-associated phosphatidylserine (PS) exposure on the outer plasma membrane was also analyzed by flow cytometry using the PI/Annexin V-FITC double staining method (kit Beckman Coulter, Villepinte, France); this method effectively distinguishes apoptotic cells from necrotic cells. Annexin V-positive and propidium iodide-negative cells were scored as apoptotic cells. Cells were analyzed with a FACSCalibur flow cytometer (Becton Dickinson, Heidelberg, Germany) acquiring 10 000 events.

### Flow cytometric analysis

Expression of TrkA, TrkB, p75^NTR^, BDNF and NGF was studied on 2×10^6^ cells by flow cytometry with or without (TrkA, TrkB and p75^NTR^) permeabilization (Intrastain Kit, DakoCytomation, Glostrup, Denmark) as per the manufacturer's instructions. After washing in 1% BSA-PBS and incubation with 5% BSA-PBS for 15 min at room temperature, cells were incubated at room temperature for 15 min with either anti-TrkA or anti-TrkB (12.5 µg/ml) or anti-p75^NTR^, anti-BDNF, anti-NGF (10 µg/ml) or isotypic controls. After washing, samples were then incubated with Alexafluor 488-conjugated goat either anti-mouse IgG Ab or anti-rabbit IgG Ab (both, 1 µg/ml; Invitrogen, Cergy Pontoise, France) for 15 min at room temperature. After washing, cells were suspended in PBS and analyzed with a FACSCalibur flow cytometer (Becton Dickinson). For samples without permeabilization (membrane expression of receptors), all incubations were realized for 30 min at 4°C and finally resuspended in PBS with 1% formaldehyde, before analysis by flow cytometry acquiring 10 000 events.

### Immunofluorescence microscopy

After two washes in PBS, samples (2–4×10^6^ cells) were fixed for 30 min at 4°C with 1% formaldehyde in PBS for 10 min at 4°C. After washing, an ice-cold 100% methanol permeabilization step was realized for 10 minutes at 4°C and then cells were rinsed in PBS for 5 min. The samples were further blocked with 5% goat serum in PBS and then incubated for 30 minutes at 4°C with primary antibodies (anti-Trk, anti-BDNF, anti-NGF, p75^NTR^) or IgG controls. After washing, cells were incubated with Alexafluor-conjugated secondary antibodies (dilution: 1/1000) for 30 min at 4°C. After two step washes, samples were finally incubated with DAPI (Molecular Probes, Invitrogen), coverslipped and sealed using mounting media (Dako North America, Carpinteria, CA, USA). Pictures were captured using a Leica microscope and a Leica digital camera. Images were processed using Leica IM500 Image Manager. For cell surface antigen stainings, incubations with primary and secondary antibodies were realized before the formaldehyde fixation step.

### Western blotting

Proteins were obtained from whole cell lysates or from supernatants of cell cultures. After two washes in PBS, cell lysates were prepared using lysis buffer (20 mM Tris–HCl [pH 7.5], 1% Nonidet P40, 10% glycerol, 150 mM NaCl supplemented with 1% protease and phosphatase inhibitor mixtures) (Sigma) followed by centrifugation at 20 000 g for 20 min at 4°C. Cell supernatants were concentrated to analyze neurotrophins released by B cultured cells. Briefly, growth medium was collected and centrifuged for 30 min at 3000 g in vivaspin columns (Millipore, Billerica, MA, USA). Equal amounts of proteins from cell lysates and cell supernatants (50 µg/lane) were separated on SDS-polyacrylamide gels under denaturing conditions and transferred onto nitrocellulose sheets (PALL Gelman Laboratory, Ann Arborn, MI, USA). Nonspecific binding sites were blocked for 1 h with 5% nonfat dry milk in TBS containing 0.1% Tween 20. After overnight incubation at 4°C with specific primary Ab (dilution 1/200), membranes were incubated with appropriate HRP-conjugated secondary Ab (DakoCytomation; dilution 1/1000) for 1 h at room temperature and revealed by an enhanced chemiluminescent detection method (Immubilon Western, Millipore). Protein-loading control was performed with anti-βactin Ab. Western blots were scanned using a bioimaging system (Genesnap; Syngene Europe, Cambridge, UK).

For co-immunoprecipitation studies, cell lysates (200 µg) were incubated with anti-Akt or anti-BDNF antibody (1/50) for 1 h at 4°C and then with protein A/G-Agarose beads (Santa Cruz Biotechnology) with gentle mixing overnight at 4°C. Beads were washed three times with lysis buffer and subsequently boiled for 5 min in SDS-sample buffer and finally immunoprecipitates were subjected to SDS-polyacrylamide gel electrophoresis, before analysis by western blotting.

### Measurement of neurotrophin secretion by ELISA

BDNF and NGF concentrations in cell supernatants were determined by using the BDNF E_max_ and NGF E_max_ ImmunoAssay Systems (Promega France, Charbonnières les Bains, France) as previously described [24]. This assay cannot distinguish between pro-NT and mature NT. When detected, neurotrophin secretions were expressed as pg/10^6^ cells.

### RT-PCR analysis

TRIzol was used to isolate total RNA from 5×10^6^ SUDHL cells. cDNA synthesis was performed with the Super Script III First-strand synthesis kit (Invitrogen) according to the manufacturer's instructions, using oligo(dT). Then amplification was performed using *Taq*DNA polymerase (Invitrogen) and specific primer sequences (Table 1) as previously reported [24]. Transcripts of NGF, BDNF, TrkA, TrkB, p75^NTR^ and sortilin were obtained using UnoCycler (VWR France, Fontenay sous Bois, France). Total RNA isolated from a human neuroblastoma cell line (IMR32) was used as a positive control.

**Table 1:** Primers used in RT-PCR studies.

Name	Primer sequences	Amplified fragments	Hybridation temperature	3′ location
**BDNF**	F: TACTTTGGTTGCATGAAGGCTGCC	266 pb	58°C	596
	R: ACTTGACTACTGAGCATCACCCTG			
**NGF**	F: ATACAGGCGGAACCACACTC	313 pb	58°C	123
	R: TGCTCCTGTGAGTCCTGTTG			
**TrkA**	F: TCAACAAATGTGGACGGAGA	197 pb	58°C	1372
	R: GTGGTGAACACAGGCATCAC			
**TrkB145**	F: AGGGCAACCCGCCCACGGAA	571 pb	60°C	2860
	R: GGATCGGTCTGGGGAAAAG			
**TrkB95**	F: GTTTCATAAGATCCCACTGGA	261 pb	58°C	7111
	R: TGCTGCTTAGCTGCCTGAGAG			
**p75^NTR^**	F: GTGGGACAGAGTCTGGGTGT	200 pb	60°C	3109
	R: AAGGAGGGGAGGTGATAGGA			
**Sortilin**	F: CTGGGTTTGGCACAATCTTT	199 pb	60°C	1161
	R: CACCTTCCTCCTTGGTCAAA			
**β-actin**	F: TGGATTCCTGTGGCATCCATGAAAC	355 pb	58–60°C	2828
	R: TAAAACGCAGCTCAGTAACAGTCCG			

F: forward; R: reverse.

### Statistical analysis

Data on cell viability were compared by ANOVA with Statview 5.0 software (Abacus Concepts). A value of *p*≤0.05 was considered as significant.

## Results

### Expression and production of NTs in DLBCL cell lines

**Figure 1:**
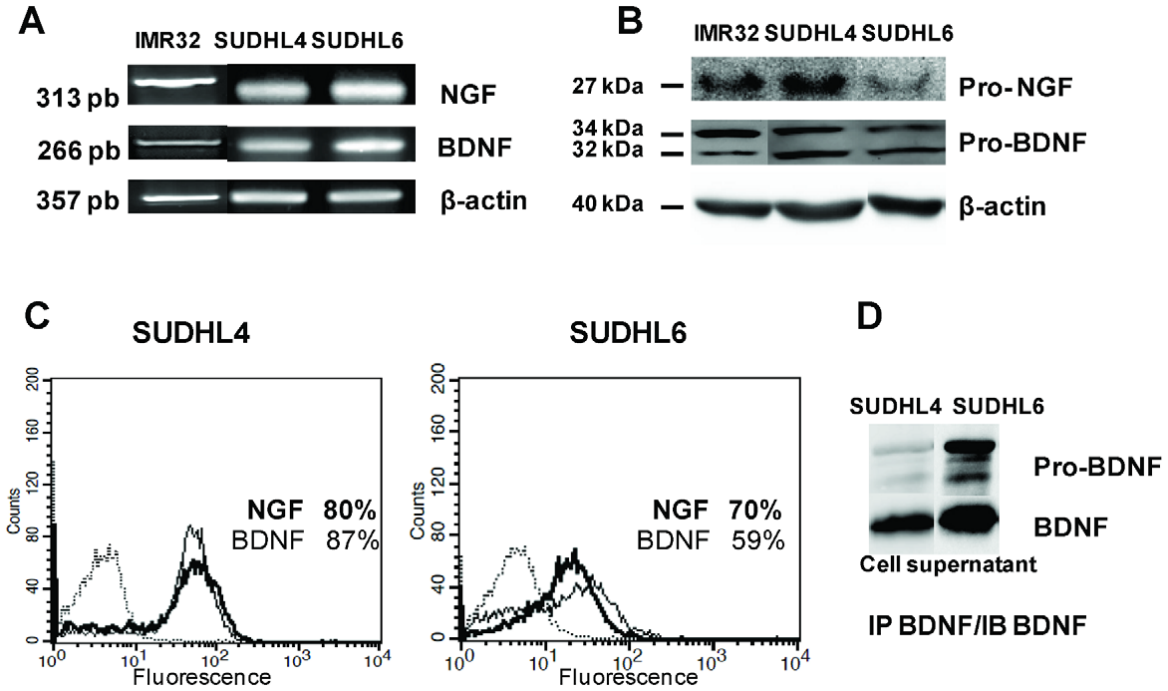
DLBCL cell lines produce neurotrophins. **A:** RT-PCR detection of NGF and BDNF mRNA was performed on SUDHL4 and SUDHL6 cells cultured with 10% FCS for 72 h. β-actin was included as a control of cDNA quality. **B:** Neurotrophin production was confirmed by western blotting of cell lysates demonstrating immature forms. Blots were reprobed with anti- β-actin as a loading control. **C:** Flow cytometry analysis demonstrating NGF (bold line) and BDNF intracellular expression in permeabilized cells (isotypic controls in dotted line). **D:** secretion of immature and mature BDNF was also detected in cell supernatants after immunoprecipitation (IP) and immunoblotting (IB) analysis. The neuroblastoma cell line, IMR32, was used as a positive control. Data are from one representative experiment out of four (RT-PCR, Flow cytometry and WB) or two (IP/WB) performed.

BDNF and NGF expression in DLBCL cell lines was characterized at the transcript level in the both cell lines SUDHL4 and SUDHL6, maintained in basal (10% FCS) culture conditions for 3 days (Figure 1A). Controls assessed that cell viability was not modified at 72 h in both SHDHL cell lines (data not shown). These expressions were confirmed at the protein level by western blotting with the detection for BDNF and, to a lesser extent for NGF, of the immature forms of NTs (pro-NGF: 27 kDa, pro-BDNF: a 32/34 kDa doublet proteins, Figure 1B). Moreover NT expression was also confirmed by flow cytometric analysis after cell permeabilization in four experiments. Indeed, as shown in Figure 1C, NGF and BDNF were detected in the majority of cells under basal culture conditions (80.4%±13.8% and 87.3%±5.6% of positive cells, respectively, for SUDHL4 and 77.6%±12.7% and 82.7%±16.8% of positive cells, respectively, for SUDHL6). Interestingly, pro-BDNF and mature BDNF were also found in culture supernatants from both cell lines, notably in SUDHL6 cell supernatants, after immunoprecipitation and western blotting (Figure 1D), whereas this basal production was often under the range of detection of the ELISA assay (data not shown). Moreover, secretion of NGF in basal culture conditions of SUDHL cell lines was not detected by immunoprecipitation nor by ELISA (data not shown).

### DLBCL cell lines express p75^NTR^/sortilin and TrkB receptors

**Figure 2:**
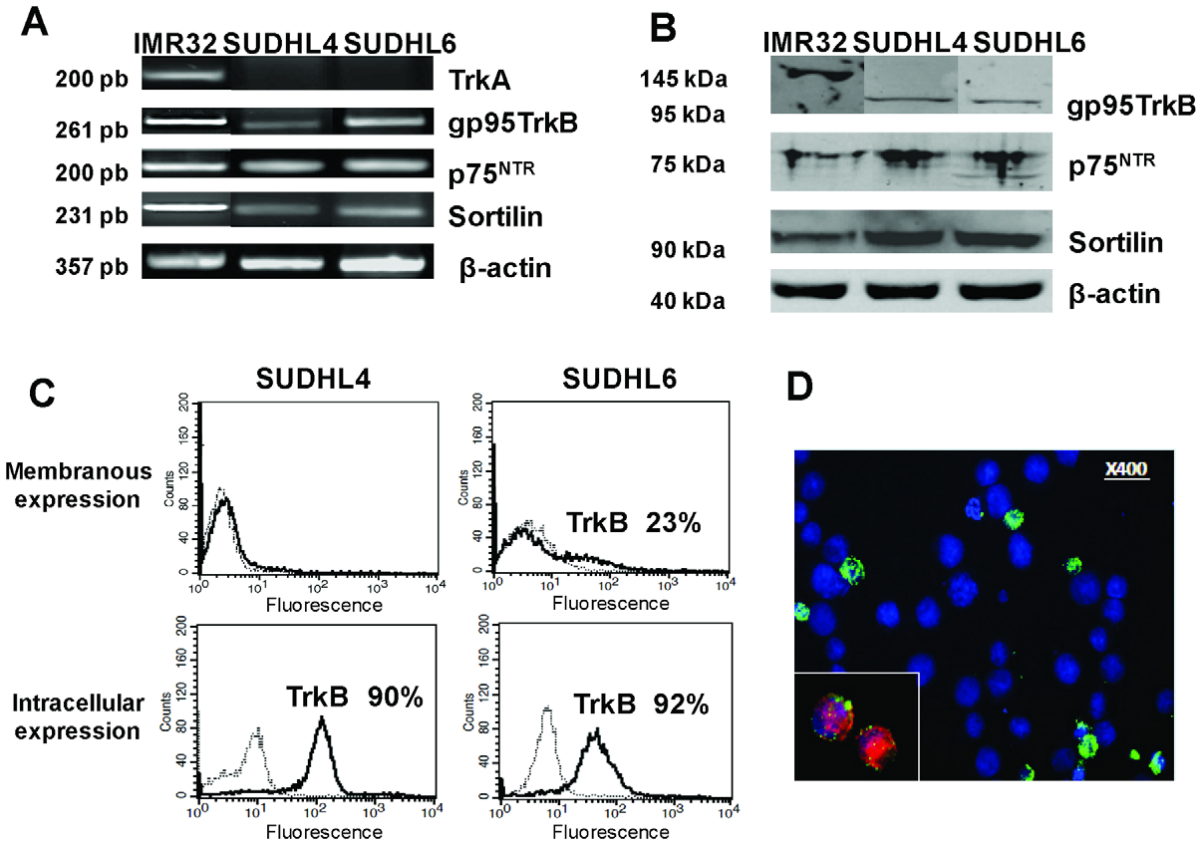
DLBCL cell lines express neurotrophin receptors. A: RT-PCR detection of p75^NTR^, and its co-receptor sortilin, TrkA and truncated gp95TrkB mRNA was performed on SUDHL cells cultured with 10% FCS for 72 h. β-actin was included as a control of cDNA quality. **B:** The truncated TrkB, p75^NTR^ and sortilin receptor protein expression was confirmed by western blotting of cell lysates. Blots were reprobed with anti- β-actin as a loading control. **C:** Flow cytometry analysis demonstrating TrkB membrane detection expressed in percentage of positive cells (bold line) in unpermeabilized SUDHL6 cells in contrast to SUDHL4 cells, whereas both cell lines expressed intracellular TrkB (dotted line: the isotypic controls). **D:** Immunofluorescence staining was performed, as described in Materials and Methods, in DLBCL cell lines that confirmed surface TrkB expression (in green) in some BDNF (inset in red) positive SUDHL6 cells (in blue: DAPI staining). The neuroblastoma cell line, IMR32, was used as a positive control. Data are representative of four independent experiments.

To evaluate the possibility of an autocrine mechanism of NT signaling we also analyzed Trk and p75^NTR^ expression in the two DLBCL cell lines using RT-PCR, western-blotting and flow cytometry. Transcripts of the low-affinity common receptor p75^NTR^, and strikingly its co-receptor sortilin were detected by RT-PCR in both cell lines (Figure 2A) which was confirmed at the protein level by western blotting (Figure 2B) and immunofluorescence (p75^NTR^, data not shown). However, membrane expression of p75^NTR^ was not detected by flow cytometry (data not shown). As shown in Figure 2A, after 72 h cell culture in basal conditions we never detected TrkA mRNA in either cell line. Interestingly, RT-PCR analyses revealed, in both cell lines and notably in SUDHL6, expression of the truncated form of TrkB (gp95) (Figure 2A) but not full-length TrkB (gp145, data not shown). However, as for p75^NTR^, we could not detect by flow cytometry membranous TrkA or TrkB protein expression in SUDHL4 cells cultured in basal conditions. In contrast, as shown in Figure 2C, flow cytometric analyses from 4 separate experiments revealed intracellular staining of TrkB in the majority of cells for both DLBCL cell lines, but TrkB membrane detection was observed only for ∼20% SUDHL6 cells (17%±7% of positive cells). According that anti-TrkB antibodies were raised to recognize the extracellular domain of both isoforms of the receptor (gp95 and gp145), our results collectively suggest that the TrkB receptor expressed in SUDHL6 cells is the truncated TrkB. Finally, positive expression of TrkB by some SUDHL6 was also confirmed by immunofluorescence assays (Figure 2D), whereas isotype control antibodies did not produce any detectable staining of the cells (data not shown). Of note, this TrkB expression by SUDHL6 was observed in cells, which were also positive for BDNF staining, suggesting a potential autocrine support for BDNF/TrkB signaling pathway (Figure 2D).

### Modulation of NT production and TrkB/p75^NTR^ expressions are induced by serum deprivation

**Figure 3:**
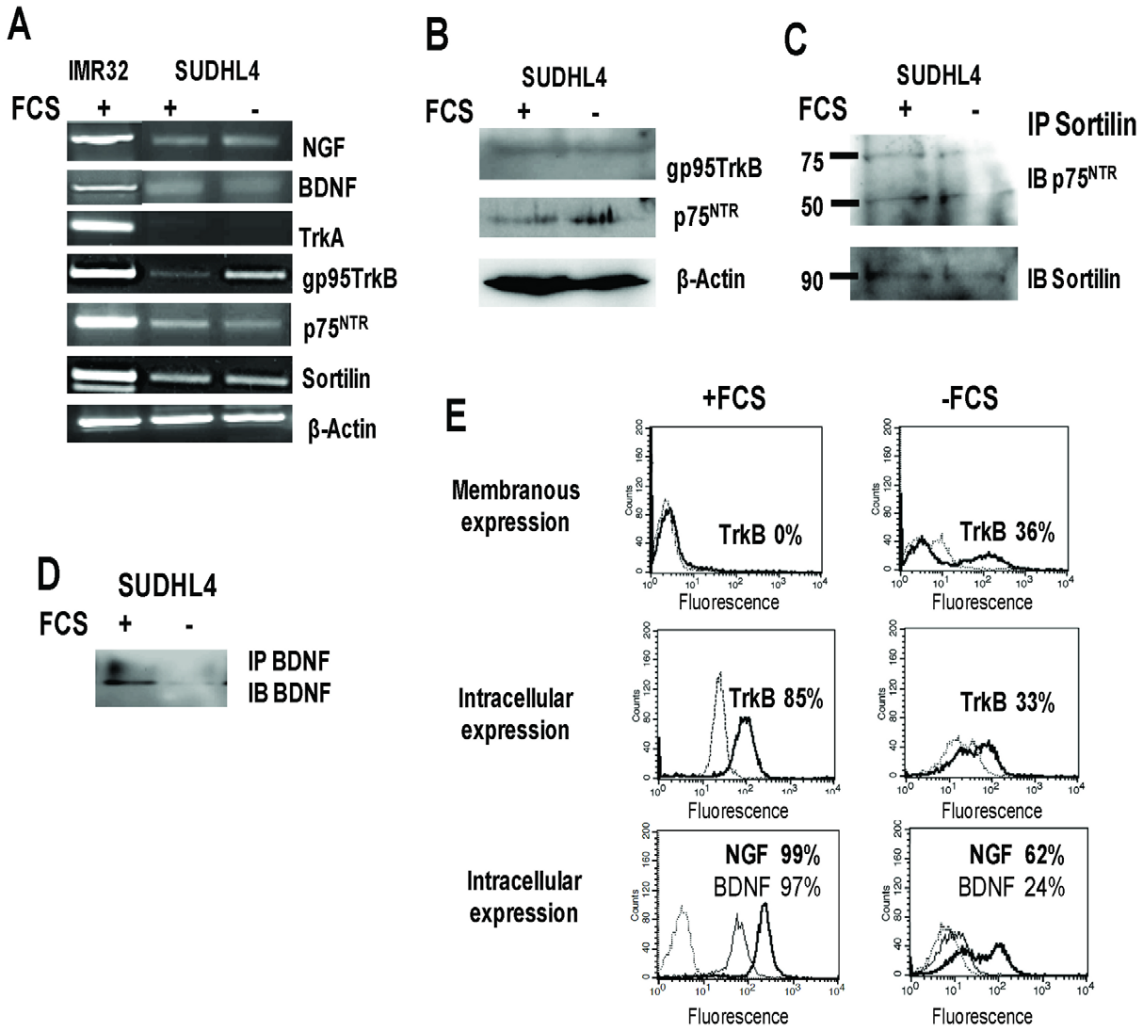
Modulation of neurotrophin and TrkB expression by SHDHL4 cells is induced by serum deprivation. **A:** RT-PCR analysis of NGF, BDNF, TrkA, gp95TrkB, p75^NTR^ and sortilin mRNA on SUDHL4 cells submitted to 72 h serum deprivation (−FCS) as compared to standard culture conditions (10% FCS, +FCS). β-actin was included as a control of cDNA quality. **B:** Western blott detection of truncated TrkB and p75^NTR^ protein expression. Blots were reprobed with anti- β-actin as a loading control. **C:** Immunoprecipitation of sortilin (IP) and western blotting analysis (IB, immunoblotting with anti-p75^NTR^ or anti-sortilin as control) demonstrating the association of sortilin with p75^NTR^ in cell lysates of both culture conditions. **D:** secretion of BDNF detected in cell supernatants after immunoprecipitation and western blotting. **E:** Flow cytometry analysis demonstrating membranous expression of TrkB (bold line) and intracellular TrkB, NGF (bold line) and BDNF expressions expressed in percentage of SUDHL4 positive cells cultured with or without FCS (dotted line: isotypic controls). Data are representative of four independent experiments.

As membranous expression of TrkB and p75^NTR^ was moderate or not present respectively in basal conditions, we further analyzed these expressions and NT production under stress conditions. Thus, cells were studied after serum deprivation for 24–72 h. Results were obtained after 72 h serum deprivation only for SUDHL4 cells, which were more resistant to this apoptotic condition. The apoptotic response was evaluated by flow cytometric detection of DNA fragmentation (27%±3% of sub-G1 cells in deprived condition, as compared to 3%±3% in the 10% FCS control, data not shown). Under such conditions, we observed a strong reduction in the number of NGF and BDNF positive SUDHL4 cells surviving after 72 h in culture (Figure 3E). Consistent with these data, production of BDNF in cell supernatant was no longer detectable after immunoprecipitation and western blotting (Figure 3D). Strikingly, after 72 h culture in FCS-free medium, mRNA TrkB truncated expression was clearly enhanced (Figure 3A) as compared with basal conditions (10% FCS), which was confirmed at the protein level by flow cytometry (Figure 3E). Indeed, in contrast to basal conditions, TrkB membranous expression appeared in 30%±10% SUDHL4 deprived cells after 72 h culture, whereas intracellular detection of TrkB was strongly reduced, suggesting membrane relocation of this receptor. Finally a weak enhanced expression of protein p75^NTR^ was also observed by western-blotting in serum deprived cell lysates, without affecting sortilin expression or association (Figure 3B and C). Of note, the bands of p75^NTR^ were detected at 75 and 50 kDa after sortilin immunoprecipitation, corresponding to the mature and the ectodomain shedding pieces of p75^NTR^ respectively. Collectively, our results showed lower neurotrophin production and enhanced expression of receptors (i.e. p75^NTR^, truncated TrkB) in association with apoptotic culture conditions.

### Rituximab sensitivity of DLBCL cell lines is associated with regulation of NT production and Trk receptor expression

**Figure 4:**
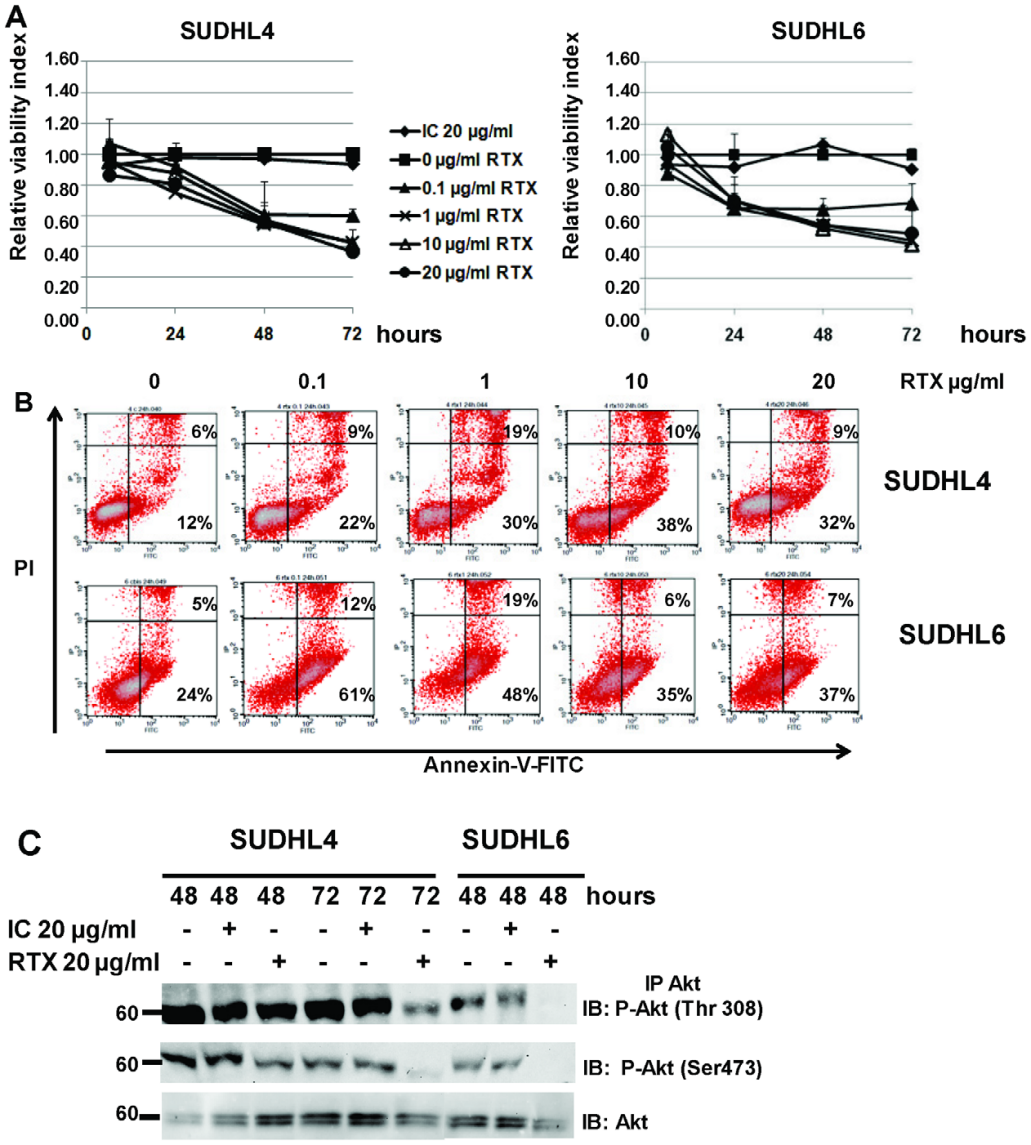
Effects of rituximab treatment on DLBCL cells lines. **A:** Viability of SUDHL4 and SUDHL6 cells exposed to various concentrations of rituximab (RTX) during 72 h in serum basal culture condition (10% FCS). Viability was determined by XTT assay and calculated relative to time-matched untreated (0) controls. A control human IgG was also used as negative control (IC: Isotype control). Results are expressed as means ± SD of four experiments. **B:** Apoptosis induced by rituximab was measured after 24 h culture by Annexin-V-FITC/PI dual staining and the ratio of apoptotic (lower right quadrant) and necrotic (upper right quadrant) cells expressed as cell percentages was analyzed by flow cytometry. **C:** Rituximab induced inhibition of PI3K/Akt signaling which was detected by immunoprecipitation of Akt and western blott analysis of phosphorylated Akt (P-Akt) at 48 h and 72 h in SUDHL6 and SUDHL4 cell lysates. Data are representative of four independent experiments.

**Figure 5:**
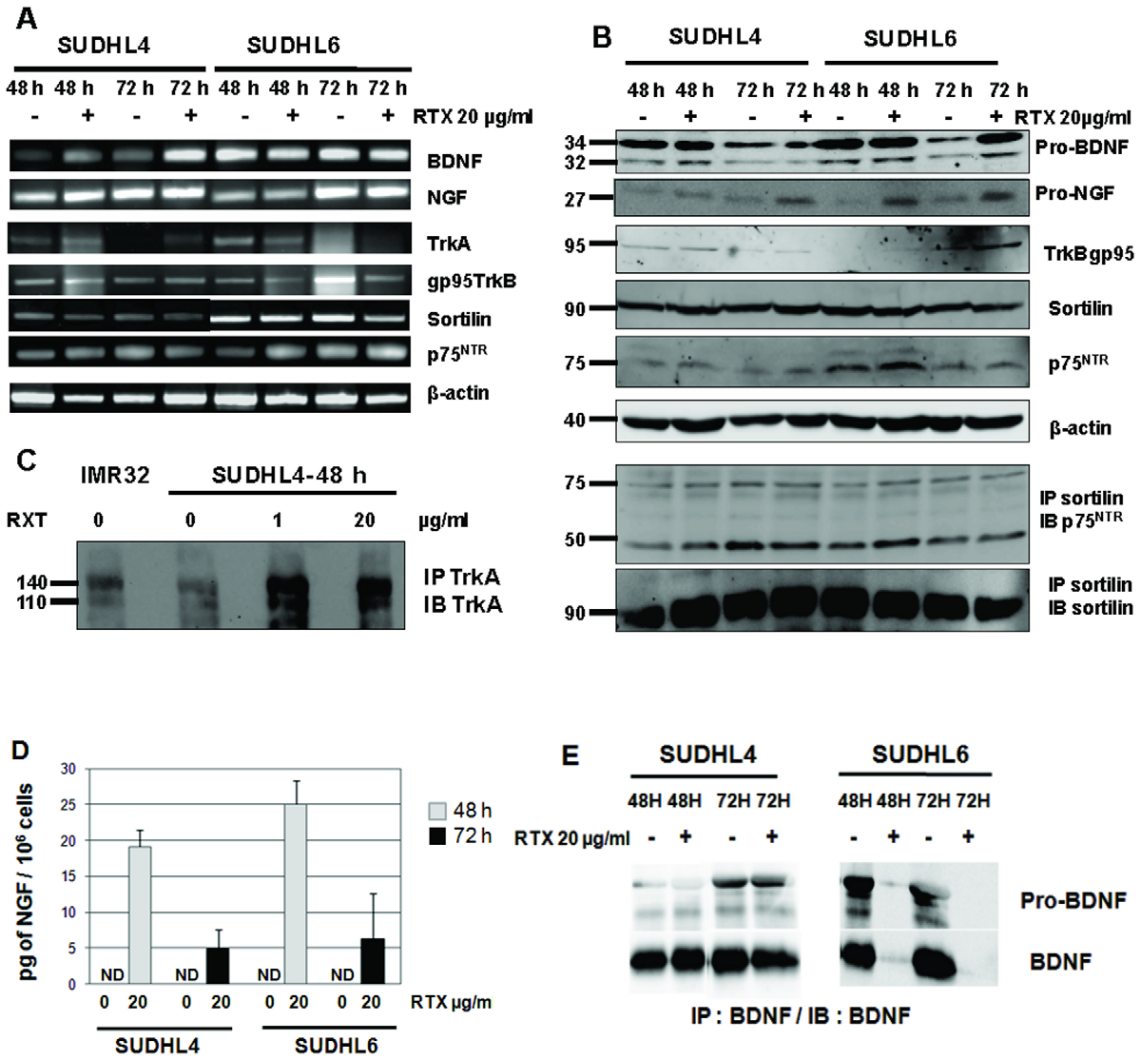
Effects of rituximab on neurotrophin production and Trk expression in DLBCL cells lines. **A:** RT-PCR analysis of NGF, BDNF, TrkA, truncated TrkB, sortilin and p75^NTR^ mRNA in SUDHL4 and SUDHL6 after 48 h and 72 h exposition to 20 µg/ml rituximab (RTX). β-actin was included as a control of cDNA quality. **B:** Western blott demonstrating in particular NGF protein expression following rituximab treatment and heterodimerization of p75^NTR^ with sortilin in cell lysates. Blots were reprobed with anti- β-actin as a loading control. **C:** Enhanced TrkA expression induced by rituximab was observed for the less rituximab sensitive cell line, SUDHL4, after immunoprecipitation (IP) and immunoblotting (IB) with specific antibodies. **D:** NGF production was confirmed after rituximab exposure by detection with ELISA in cell supernatants of both cell lines, whereas it was undetectable (ND, non detected) in control cultures. Results are means ± SD of three independent experiments. **E:** BDNF secretion seemed to decrease in the most rituximab sensitive cell line, SUDHL6, as shown by western blott analysis of BDNF in cell lysate immunoprecipitates. Data are from one representative experiment out of four (RT-PCR, WB) or two (IP/WB) performed.

The CD20-expressing human DLBCL cell lines tested in the present study has been reported to have differential apoptotic sensitivity in response to rituximab, SUDHL6 cells being more sensitive than other DLBCL cell lines and notably SUDHL4. Thus to evaluate a potential link between anti-CD20 sensitivity and NT production, we analyzed secretion of NGF and BDNF, as well as expression of receptors in cell cultures with or without rituximab. As a negative control, an isotype control of rituximab was also done (IC). To investigate the dose-response relationship and the kinetics of growth inhibition and cell death induced by rituximab, SUDHL cells were incubated in the presence of various concentrations of rituximab (0, 0.1, 1, 10 and 20 µg/ml) for 6 to 72 h. Cell viability was then monitored by the XTT assay. Furthermore, the ability of rituximab to induce apoptosis in SUDHL cells was measured by detecting PS translocation using Annexin V/PI staining, and evaluation of DNA fragmentation, followed by flow cytometry analyses. Figure 4 shows results obtained (viability) or representative (annexin V/PI stainings) of 4 independent experiments. Viability and PS translocation data confirmed the apoptotic effect of rituximab on both DLBCL cell lines, with a dose-dependent and a kinetic effect. No significant difference was observed when the isotypic control was compared to the culture control without rituximab (Figure 4A). Furthermore, statistical analysis of the rituximab effect revealed by the XTT test showed a significant reduction of viability, which was seen for both cell lines from 1 µg/ml rituximab and after 48 h treatment (i.e. at 1 µg/ml rituximab: *p*<0.01 and *p*<0.05 for SUDHL4 and SUDHL6 respectively). This effect was enhanced after 72 h of cell exposure (*p*<0.001 for both cell lines and from 1 µg/ml rituximab). However, the percent of hypodiploid nuclei (sub-G1) induced by rituximab in the two cell lines was relatively low, even if a pronounced effect was found with SUDHL6 (data not shown). The maximum response was reached for SUDHL4 at 20 µg/ml rituximab after 72 h (Figure 4A). In contrast, SUDHL6 cells were more sensitive, with a strong cell cytotoxicity following 24 h exposure to rituximab, which was observed even at low dose of rituximab (*p* = 0.05 for 0.1 and 1 µg/ml rituximab treatment of SUDHL6 cells, whereas no significant effect was observed with the SUDHL4 cell line). This differential apoptotic sensitivity of the two cell lines in response to rituximab was confirmed by flow cytometric analysis. A dose response was obtained for SUDHL4 after 24 h exposure, whereas a strong apoptotic response was often observed in SUDHL6 at this time for lower doses of rituximab (i.e. 61% of apoptotic cells as shown in Figure 4B), following by a decrease with high concentrations. Consistent with this, constitutive phosphorylation of Akt seemed to be enhanced in the more rituximab-resistant cell line, SUDHL4. Moreover, inhibition of the constitutively active PI3K/Akt signaling pathway was observed in SUDHL6 cells previously than SUDHL4 cells. Indeed, in contrast to the weak inhibition of P-Akt (Ser473) observed in SUDHL4, a strong dephosphorylation of Akt (Ser473 and Thr308) by rituximab was detected in SUDHL6 cell lysates after 48 h exposure to 20 µg/ml rituximab (Figure 4C). As results for NTs strongly suggested an autocrine/paracrine survival axis in the SUDHL cell lines tested, we next analyzed expression of NTs and their receptors in cell cultures after 48 and 72 h exposure to rituximab (20 µg/ml). We found that rituximab induced NGF expression in both cell lines (Figure 5B), which was associated with the detection of NGF in cell supernatants. In contrast to basal culture conditions, strong NGF secretion was detected by ELISA for both cell lines after 48 h rituximab exposure that further decreased at 72 h (Figure 5D). In SUDHL4, the less rituximab sensitive cell line, it is noteworthy that NGF secretion was correlated with TrkA receptor expression, although weak, both at the transcript and protein levels, in contrast to SUDHL6 cells. Indeed, a low level of TrkA mRNA was detected after 48 h (SUDHL4 and SUDHL6 cells) and 72 h (SUDHL4 cells) of rituximab exposure (Figure 5A), but enhanced expression of the TrkA receptor at the protein level was only observed for SUDHL4 cells by immunoprecipitation of TrkA in cell lysates (Figure 5C). As shown in Figure 5A,TrkA mRNA were also slightly detected after 48 h of culture in the control cell of both cell lines. Furthermore, we observed a decreased production of BDNF and pro-BDNF in cell supernatants of the most rituximab sensitive cell line, SUDHL6, exposed to rituximab, in contrast to SUDHL4 cells which seemed to maintain BDNF synthesis (Figure 5E). Moreover, rituximab did not modify expression of the truncated form of TrkB (gp95), or p75^NTR^ or sortilin expression at both mRNA and protein levels (Figure 5 A and B). Similarly, there was no change in protein expression of the p75^NTR^ forms (75 and 50 kDa) found in sortilin immunoprecipitates in both cell line lysates. Thus, these data suggest that differences in rituximab-induced apoptoptic sensitivity could involve differential expression of survival Trk signaling in DLBCL cell lines.

### Pharmacologic inhibition of Trk receptors induces apoptosis of DLBCL cells

**Figure 6:**
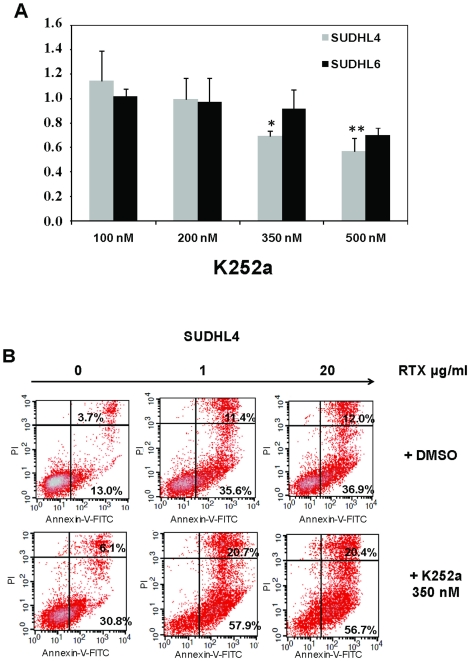
Pharmacologic inhibition of Trk receptors inhibits survival of DLBCL cell lines and synergizes rituximab induced apoptosis. A: Cell viability was evaluated using the XTT test for both SUDHL cell lines cultured for 24 h in presence of various concentrations of K252a. Data are expressed as means ± SD of the relative cell viability of untreated control cells obtained from three independent experiments. Significant *p* values (*: *p*<0.05 and **: *p*<0.01) were determined in comparison with 100 nM K252a. **B:** K252a (350 nM) induced apoptosis in SUDHL4 cells, demonstrating a synergistic effect with rituximab (0–20 µg/ml) exposure. Example of flow cytometry analysis of apoptosis using Annexin-V-FITC and propidium iodide (PI), as detailed in legend of Figure 4, obtained after 48 h cell culture. Data are from one representative experiment out of three performed.

Presence of both Trk receptors and NTs, notably BDNF expressed with TrkB receptors, in both cell lines suggest an autocrine pathway of neurotrophin signaling in DLBCL. In order to address the functionality of Trk receptors expressed in DLBCL cell lines, SUDHL cells were incubated with various concentrations of the Trk inhibitor K252a and cell survival was measured (Figure 6A). Results showed a strongest sensitivity of SUDHL4 cells to K252a by 24 h treatment, with significant decrease of viability correlated with increasing amounts of K252a. Indeed, SUDHL6 cells were significantly sensitive to cell death after 48 h of K252a exposure (data not shown). As shown in Figure 6B, K252a-induced cytotoxicity in SUDHL4 cells was associated with a strong apoptosis, measured with annexin-V/PI staining, after a 48 h exposure (i.e. 30.8% of apoptotic cells with 350 nM K252a *vs* 13% in DMSO culture controls). Interestingly, apoptosis obtained in SUDHL4 cells by inhibiting Trk receptors was comparable to the apoptotic effect of rituximab (i.e. 36.9% of apoptotic cells for 20 µg/ml rituximab). Furthermore, K252a exhibited additive cytotoxic effects with rituximab (i.e. 56.7% of apoptotic cells in presence of rituximab 20 µg/ml+K252a 350 nM). These data strongly support our hypothesis that autocrine neurotrophin signaling is an important survival factor for DLBCL cell lines, and may interfere with rituximab leading to therapeutic resistances.

## Discussion

In the present study we demonstrated that DLBCL cell lines expressed the gene transcripts and proteins for BDNF and to a lesser extent NGF, and the high-affinity truncated TrkB as well low-affinity p75^NTR^ receptors. Furthermore, secretion of BDNF and pro-BDNF were also observed in DLBCL cell line supernatants, even though variable with the cell line. Moreover, in both cell lines, we detected strong sortilin (NTR3) expression, an intracellular transport protein for NTs and proNTs which is also involved, by heterodimerization with p75^NTR^, in the cell death effect of pro-NTs. However, they lacked, in basal culture conditions, detectable mRNA expression for the full-length TrkB and TrkA receptors, but expressed the truncated form of TrkB. NT production and Trk receptor expression, including TrkA, seemed to be regulated by culture conditions that modulate cell survival (serum deprivation or rituximab exposure).

Several microarray studies performed on untreated *de novo* DLBCL identified two main prognostically different subgroups. Both were characterized by a distinct gene expression profile either characteristic of normal germinal center B-cells or activated blood memory B-cells. The germinal center B-cell-like (GCB) subgroup was correlated with a significantly better prognosis in comparison to the activated B-cell-like (ABC) subgroup [27], [28]. mRNA for NGF and BDNF were recently detected in ABC (OCI-LY3) and GCB (OCI-LY19) DLBCL cell lines, but NT protein production was undetectable by ELISA [23]. In the present study we used two GCB DLBCL cell lines (SUDHL4 and SUDHL6) [27], [29]. We confirmed previous data and further demonstrated for the first time that NT secretion (BDNF) can be observed in cell supernatants after basal culture conditions (10% FCS). Of note, BDNF production was associated with a strong cytoplasmic TrkB sequestration suggesting, as was demonstrated in B cell lines [24], a potential survival autocrine signaling pathway. On the other hand, as shown by coimmunoprecipitation and western blotting analyses, association of sortilin with the death receptor p75^NTR^ was also observed, for the first time in DLBCL cells, in the present study. Moreover, we detected also the ectodomain shedding pieces (50 kDa) of p75^NTR^ in the sortilin immunoprecipitates. These data suggest proteolytic processing of p75^NTR^ and thus release of the intracellular domain of the receptor that is known to occur after ligand activation, initiating a second signaling apoptosis step [30]. Proteolytic processing of p75^NTR^, together with strong intracytoplasmic sequestration of the receptor, may explain our difficulties in membrane detection of this receptor by flow cytometry. Indeed, the anti-p75^NTR^ used for this analysis was selected for its ability to target the extracellular domain of human p75^NTR^. Present data demonstrated that pro-NTs, and notably pro-BDNF, are produced by both DLBCL cell lines tested. As the heterodimeric receptor p75^NTR^/sortilin is known to be required for the cell death effect of pro-BDNF [13], [31], pro-BDNF-p75^NTR^/sortilin signaling pathway may act, in the absence or low membrane TrkB expression, by sensitizing cells to apoptotic cell death.

We detected expression of the truncated form of TrkB (gp95) but not full-length, in both DLBCL cell lines. Of note, the prominent form of truncated TrkB mRNA was previously reported in normal B lymphocytes [24]. The putative function of these truncated TrkB receptors, lacking tyrosine kinase activity, is still not clear. However, they can exert a functional activity in neurons, allowing activation and proliferation of neuroblasts in the presence of BDNF [32], [33]. Moreover, in the present study, gp95TrkB expression increased in DLBCL cell line (SUDHL4) under stress conditions (i.e. serum deprivation), with a membrane relocation of the receptor as for previous B cell lines [24]. In contrast to TrkB, the heterodimeric p75^NTR^/sortilin death receptor seemed to be unaffected by serum deprivation. This TrkB receptor relocation may suggest an autocrine survival mechanism of neurotrophin signaling in deprived cells, as we recently demonstrated in colorectal cancer cells [34]. However, we also observed a strong decrease in BDNF production in association with apoptosis which argues for a potential survival function of BDNF. Pharmacological inhibition of Trk receptors (K252a, 200 nM) was recently shown to inhibit proliferation and survival of malignant B cells, including an ABC DLBCL cell line (OCI-LY3) whereas the truncated TrkB receptor was the major Trk receptor expressed by these cells [23]. But an apoptotic effect of K252a was not observed on the GCB cell line tested (OCI-LY19). In the present study, we showed a differential response of the two GCB cell lines used. Whereas K252a significantly decreases the cell viability in DHL4 cells, it did not alter the viability of DHL6 cells at least after 24 h drug exposure, while cytotoxic effect was only observed by 48 h and for higher drug concentrations (500 nM, data not shown). Such differences in data can be explained by variations in the Trk receptors expressed in DLBCL cell lines. In addition to the presence of TrkB transcripts in both cell lines, TrkA mRNA was also detected in the previous report of Sniderhan *et al.* [23] for OCI-LY3 cells and not for OCI-LY19 cells suggesting potential TrkA and TrkB protein expression in the ABC cell line as compared to GCB. In our study, albeit we did not detect TrkA protein expression in basal culture conditions in both cell lines, we had observed a low level of TrkA mRNA after 48 h standard culture conditions, suggesting a possible regulation of TrkA expression by the cell cycle. Although K-252a affects several other kinases at higher concentrations and the effects observed may thus not only be due to inhibition of Trk signaling [35], numerous studies have used nanomolar quantities of K252a as a selective inhibitor of NT signaling. Thus, our present data strongly support the notion that SUDHL cells express functional Trk receptors.

The exact mechanism of rituximab resistance, a growing concern, is not entirely clear. Several tumor-associated and host-associated mechanisms have been proposed, including mutations or poor surface CD20 antigen expression and, more recently, polymorphisms in FcγRIIIA receptor [36]. To further assess the role of NT production in rituximab sensitivity, we exposed cells to rituximab, as the two studied SUDHL cell lines have been reported to have differential apoptotic sensitivity in response to rituximab [26]. As expected, apoptosis induced by rituximab was observed in both cell lines and associated with an inhibition of the constitutively activated PI3K-Akt signaling pathway, known to lead to chemosensitization [5], [37]. Furthermore, we confirmed the higher apoptotic sensitivity of SUDHL6 [26]. Such differential sensitivity to apoptosis may be explained by the lower constitutively expression of P-Akt detected in SUDHL6 cells as compared to SUDHL4, in accordance to previous observations for other DLBCL cell lines [5]. Indeed, we did not find a significant difference in CD20 expression between the two cells lines (data not shown), as was previously reported [38]. Strikingly, we demonstrated for the first time that NGF production and secretion in cell supernatants were induced by rituximab exposure in both cell lines SUDHL4 and SUDHL6, by contrast to the weak NGF expression detected in basal culture conditions. Furthermore, up-regulation of TrkA expression was observed after 48 h exposure to rituximab in SUDHL4 cells, and not in the more sensitive cell line SUDHL6. In addition, rituximab-induced apoptosis was concomitantly associated with a decreased of BDNF secretion in SUDHL6 cell supernatants. These results argue for a relationship between rituximab resistance and an autocrine NT survival loop through Trk receptor signaling of survival pathways, inhibited by rituximab. This hypothesis was supported by pharmacologic Trk inhibition (K252a) that enhanced rituximab-induced apoptosis of SUDHL cells. While exposure of SUDHL4 cells to K252a alone resulted in apoptotic cell death comparable to the effect induced by rituximab, the combination of both was synergistic in the apoptotic response of the more resistant DLBCL cell line. These data suggest that a NT/Trk fine-tuning regulation may be of crucial important in drug sensitivity/cell survival in DLBCL. However, mechanisms underlying these differential regulations of NT/Trk expression induced by rituximab in SUDHL cells remains to be clarified.

In conclusion, therapeutic targeting of Trk-neurotrophin axis is beginning to emerge for NHL and present results point out to this possibility in DLBCL. We demonstrated notably that secretion of NGF, BDNF and the expression of their survival Trk receptors are regulated in DLBCL cell lines by culture conditions, and potentially could contribute to malignant cell survival and rituximab resistance.
